# A computational method for predicting nucleocapsid protein in retroviruses

**DOI:** 10.1038/s41598-021-03182-2

**Published:** 2022-01-11

**Authors:** Manyun Guo, Yucheng Ma, Wanyuan Liu, Zuyi Yuan

**Affiliations:** 1 Cardiovascular Department, The First Affiliated Hospital of Xi’an Jiaotong University, No. 277 W. Yanta Road, Xi’an, 710061 Shaanxi People’s Republic of China; 2 School of Electronics & Control Engineering, Chang’an University, Middle Section of Nan Er Huan, Xi’an, 710064 Shaanxi People’s Republic of China

**Keywords:** Bioinformatics, Machine learning, Computational biology and bioinformatics, Protein sequence analyses

## Abstract

Nucleocapsid protein (NC) in the group-specific antigen (*gag*) of retrovirus is essential in the interactions of most retroviral *gag* proteins with RNAs. Computational method to predict NCs would benefit subsequent structure analysis and functional study on them. However, no computational method to predict the exact locations of NCs in retroviruses has been proposed yet. The wide range of length variation of NCs also increases the difficulties. In this paper, a computational method to identify NCs in retroviruses is proposed. All available retrovirus sequences with NC annotations were collected from NCBI. Models based on random forest (RF) and weighted support vector machine (WSVM) were built to predict initiation and termination sites of NCs. Factor analysis scales of generalized amino acid information along with position weight matrix were utilized to generate the feature space. Homology based gene prediction methods were also compared and integrated to bring out better predicting performance. Candidate initiation and termination sites predicted were then combined and screened according to their intervals, decision values and alignment scores. All available *gag* sequences without NC annotations were scanned with the model to detect putative NCs. Geometric means of sensitivity and specificity generated from prediction of initiation and termination sites under fivefold cross-validation are 0.9900 and 0.9548 respectively. 90.91% of all the collected retrovirus sequences with NC annotations could be predicted totally correct by the model combining WSVM, RF and simple alignment. The composite model performs better than the simplex ones. 235 putative NCs in unannotated *gag*s were detected by the model. Our prediction method performs well on NC recognition and could also be expanded to solve other gene prediction problems, especially those whose training samples have large length variations.

## Introduction

Retroviruses encompass by a large family of infectious agents which could be categorized into seven genera according to their morphological and biochemical features^1^. Group-specific antigen (*gag*) is the genetic material that codes for the core structural proteins of a retrovirus^2^. *Gag* proteins usually contain three major domains: matrix protein (MA) at their N-terminus; capsid protein (CA) in the middle; and nucleocapsid protein (NC) at or near their C-terminus^3^. The NC domain of *gag* is essential in the interactions of most retroviral *gag* proteins with RNAs^4^. After the releasing of NC from *gag*, it participates in a wide variety of protein-RNA interactions. Many of them involve its nucleic acid-chaperone activity. The NC domain is a key component of the assembly processes because it is required for the recognition and packaging of the RNA genome^5^ and also responsible for binding to the RNA scaffold^6^.

The indispensable role of NC attracts many researchers. Evidence that a central domain of NC is required for RNA packaging in murine leukemia virus were found^7^. Arginine methylation of the HIV-1 NC were found to result in its diminished function^8^. NAC activity of HIV-1 NC was found to play a critical role in reverse transcription and its molecular mechanism was studied^9^. Inhibitors of HIV nucleocapsid protein zinc fingers were considered as candidates for the treatment of AIDS^10^.

However, the amount of NCs in retroviruses annotated by experimental method is still small (less than a hundred). Computational method could help to predict more NCs in retroviruses thus benefit subsequent structure analysis and functional study on them. A computational method to identify reading frames of human endogenous retroviruses (*gag*s included) has been proposed^11^. A platform independent tool named RetroTector that could predict conserved motifs of retroviruses was developed^12^. However, no computational method that could predict the precise locations of NCs’ initiation and termination sites has been proposed yet. Besides, length of NCs from different retroviridae genera varies from 48 to 126aa according to records in National Center for Biotechnology Information (NCBI), thus gene prediction methods for genes with conserved lengths are not applicable. Furthermore, classical database search tools^13–15^ couldn’t achieve satisfying results in prediction of retrovirus genes with large length variation^16^. Therefore, there is an urgent need to come up with a computational model for NC prediction.

In this paper, computational models to identify NCs from retroviruses were proposed. All available annotated NC sequences in retroviruses were collected for the training and testing process. Position weight matrix (PWM) along with all six parameters of factor analysis scales of generalized amino acid information (FASGAI)^17^ were used to generate the feature space for NC prediction. The initiation and termination sites of NCs were separately predicted and combined together afterwards to acquire high prediction accuracy when dealing with sequences that are poorly conserved in their lengths. Their performance was tested by fivefold cross validation test. A composite ab initio model to predict intact NCs from genetic sequences was then proposed. It performs better than the simplex ones. All of the 6651 available *gag* sequences without NC annotations were scanned with the composite model and 282 putative NCs in them were found.

## Materials and methods

### NC collection

All available amino acid sequences of retroviruses with their NCs annotated based on experimental evidences were collected from NCBI at http://www.ncbi.nlm.nih.gov. There are 77 of such sequences in total. Among them, 4 of them are beta-retrovirus, 13 of them are gamma-retrovirus, 9 of them are from delta-retrovirus, 2 of them are epsilon-retrovirus and the other 49 of them are lentivirus. All these sequences were used for the following training and testing process. All of them are with intact NC structures (please refer to S1 File for details).

### Separate prediction of NC boundaries

Traditional gene predicting methods could performance well when predicting gene sequences with fixed lengths. However, when it comes to gene sequences with large length variations, such methods may lose effectiveness or even feasibility. This might be because the constant dimension of feature space used in traditional methods couldn’t represent features of such genes properly. The lengths of annotated NCs in retroviruses range from 48 to 126aa, so an approach to revise the traditional gene predicting methods to fit the NC predicting problem is needed.

Our predicting method focuses more on the border areas adjacent to the start and end of NCs instead of interior areas away from them, for the former contain more effective information for gene prediction and are usually more conservative. The fixed length flanking residues of the initiation site and termination site were predicted to deduce the precise locations of the start and end of NCs. Initiation site and termination site were predicted separately, and the sequence between them were regarded as a candidate NC sequence only when it’s length is reasonable. Then the most probable candidate NC was singled out among all candidate NCs to be the final putative NC according to the decision value and alignment score involving it. This technique brings out both feasibility and high accuracy.

### Sample preparation

Two sets of training samples for initiation and termination sites prediction respectively were built separately. The training samples for initiation sites could be denoted as:

1 $$\left\{ \begin{aligned} & I_{p} = gag(i:i + L_{is} - 1) \hfill \\ & I_{n} = gag(i + osi:i + L_{is} - 1 + osi) \hfill \\ \end{aligned} \right.{ ,}$$

where $$i = Init(NC)$$, $$- 50 \le osi \le 50\,\& \,osi \in Z \,\& \,osi \ne 0$$.

Similarly, the training samples for termination sites could be denoted as:

2 $$\left\{ \begin{aligned} & T_{p} = gag(j - L_{ts} + 1:j) \hfill \\ & T_{n} = gag(j - L_{ts} + 1 + ost:j + ost) \hfill \\ \end{aligned} \right.{ ,}$$

where $$j = Term(NC)$$, $$- 50 \le ost \le 50\& ost \in Z\& ost \ne 0$$.

Here, $I_{p}$ denotes a positive training sample of initiation site generated from a *gag* sequence, $I_{n}$ denotes a negative training sample of initiation site. Similarly, $T_{p}$ denotes a positive training sample of termination site and $T_{n}$ denotes a negative training sample of termination site. $Init(NC)$ and $Term(NC)$ represent the true initiation site and termination site of a NC sequence. $osi$ and $ost$ are randomly generated offsets added to initiation and termination site locations respectively to generate negative samples. $L_{is}$ and $L_{ts}$ denote the length of initiation samples and termination samples.

We generated the negative sample set with a size 5 times as large as the positive sample set and took the imbalanced sample sets problem into our consideration in the modelling process, to overcome the difficulty of the lack of positive training samples.

### Feature selection

A hybrid feature space construction approach was proposed by combining position characteristics and physicochemical properties of sequences.

### Position characteristics

The widely recognized PWM^18^ was applied to extract the position characteristic of sequences. By aligning residues starting from initiation sites or ending at termination sites of positive NC sequences, PWMs are defined as follow:

3 $$M_{k,j}^{PWM} = \log (f_{kj} /b_{k} + 1) \, {.}$$

Here, $f_{kj}$ stands for the absolute frequency of amino acid $k$ in the $j{\text{th}}$ position of $N$ aligned sequences of length $l$, $j \in (1,...,l)$ , $k$ is the set of amino acids,  $b_{k} = 1/\left| k \right|$ ($\left| k \right|{ = }20$ for amino acids, so $b_{k} = 0.05$).

After generating the PWM, the position characteristic of any $l$-aa-long sequence $V$ was extracted by the following mapping method. Each amino acid of $V$ was assigned with its corresponding value in the matrix according to its position. Then a $l$-dimension-vector $V_{{}}^{Pos}$ was generated to represent the position characteristic of the original $l$-aa-long sequence:

4 $$V_{j}^{Pos} = M_{k,j}^{PWM} { ,}$$

where $$j \in (1,...,l)$$, $$k = V_{j}$$.

### Physicochemical properties

All 6 parameters of the FASGAI^19^ were selected to extract the physicochemical properties of sequences (Please refer to S2 File for details of FASGAI). FASGAI involves hydrophobicity, alpha and turn propensities, bulky properties, compositional characteristics, local flexibility, and electronic properties derived from 335 property parameters of 20-coded amino acids. Thus when dealing with an $l$-aa-long sequence, the sequence was mapped into a $6 \times l$ matrix to represent its physicochemical properties.

After combining the position characteristics and physicochemical properties, a feature space with $(1 + 6) \times l$ features in total was established for the $l$-aa-long sequence.

### Binary classifiers

In our previous study, three binary classifiers based on different principles were applied to the same feature space to test and compare their predicting abilities: weighted support vector machine (WSVM), random forest (RF) and weighted extreme learning machine (WELM). And we found that the combination of the first two of them could generate the best predicting performance^20^. Prediction models based on WSVM and RF were separately built to predict the initiation and termination sites of NCs.

### Finding candidate NCs

After the probable NC start and end locations were predicted, a combination method to combine them is required. As there may be several possible NC start and end combination pairs in one unannotated *gag* sequence, it is necessary to dispose all the less probable putative combinations and leave the most probable one as the final prediction result. The details of such ruling out strategy are shown as follow:

*Step 1*: Keep all the putative NC boundary pairs generated from RF models which have interval distance within the range of NC sequence lengths as candidate NC boundary pairs. For the $m{\text{th}}$ and $n{\text{th}}$ amino acids in a *gag* sequence, the amino acid pair $(m,n)$ is a candidate NC pair only when it satisfies:

5 $$\left\{ \begin{aligned} & NC_{\min } - e_{\min } \le n - m \le NC_{\max } + e_{\max } , \hfill \\ & C_{RFI} (S,m,L_{is} ) = 1, \hfill \\ & C_{RFT} (S,n,L_{ts} ) = 1 \hfill \\ \end{aligned} \right. \, {.}$$

where $NC_{\min }$ and $NC_{\max }$ are the minimum and maximum lengths of annotated NCs respectively, $e_{\min }$ and $e_{\max }$ are natural numbers and act as the relaxation parameters for the minimum and maximum NC lengths respectively. $L_{is}$ and $L_{ts}$ denotes the length of initiation samples and termination samples.$C_{RFI}$ and $C_{RFT}$ are Boolean variables, their values indicate whether the $m{\text{th}}$ and $n{\text{th}}$ amino acids of *gag* sequence $S$ are candidate initiation site and termination site respectively according to the prediction results from random forest models.

*Step 2*: Calculate the products of decision values of initiation and termination sites of all candidate NC boundary pairs sorted out in step 1. Then keep the candidate boundary pair with the largest product as the putative NC (A decision value is generated from WSVM models according to the distance of a sample to the classification hyper plane. The prediction result is more likely to be positive when the decision value is larger, vice versa.). Consider amino acid pair $(m,n)$ as a putative NC pair only when it also satisfies:

6 $$\mathop {\arg \max }\limits_{m,n} D_{WSVMI} (S,m,L_{is} ) \cdot D_{WSVMT} (S,n,L_{ts} ){ ,}$$

where $D_{WSVMI} (S,m,L_{is} )$ and $D_{WSVMT} (S,n,L_{ts} )$ are decision values assigned to the $m{\text{th}}$ and the $n{\text{th}}$ amino acids of *gag* sequence $S$ after computation of the WSVM models. $(m,n)$ also satisfies the constraints in (6).

### Combination with homology based method

After the screening process, the putative NCs generated by WSVM & RF models are compared with putative NCs generated from homology based methods. The results are then combined together to enhance the prediction performance. First we introduce a simple alignment (SA). Thus the locations of the putative initiation site and termination site are shown as follow:

7 $$\left\{ \begin{aligned} & \mathop {\arg \max }\limits_{m,n} D_{P} , \, if\,\,\max D_{P} /\alpha \ge \max A_{P} \hfill \\ & \mathop {\arg \max }\limits_{p,q} A_{P} , \, if\,\, \max D_{P} /\alpha < \max A_{P} \hfill \\ \end{aligned} \right. \, {.}$$

Here $\max D_{P} = \max D_{WSVMI} (S,m,L_{is} ) \cdot D_{WSVMT} (S,n,L_{ts} )$ ($m$ and $n$ also satisfy the constraints shown in (6), $\max A_{P} = \max A_{I} (S,p,L_{is} ) \cdot A_{T} (S,q,L_{ts} )$, subject to

8 $$\left\{ \begin{aligned} & A_{I} (S,p,L_{is} ){\text{ = max}}Align(S(p,p + 1,...,p + L_{is} - 1),I_{p} )/L_{is} \hfill \\ & A_{T} (S,q,L_{is} ){\text{ = max}}Align(S(q - L_{ts} + 1,q - L_{ts} ,...,q),T_{p} )/L_{ts} \hfill \\ \end{aligned} \right. \, {.}$$

Here $A_{I} (S,p,L_{is} )$ is the maximum alignment score generated from a $L_{is}$ long subsequence starting from the $p{\text{th}}$ amino acid of *gag* sequence $S$ after comparing it with all the positive training samples of initiation sites. Analogously, $A_{T} (S,q,L_{is} )$ is the maximum alignment score of a $L_{ts}$ long subsequence ending the $q{\text{th}}$ amino acid of $S$. The alignment function $Align$ calculates the total number of identical amino acids at the same locations in two sequences with equal length. Since the products of decision values of totally correct boundary pairs are close to 1 but couldn’t reach it, while the products of alignment scores have a maximum value of 1, parameter $\alpha$ is introduced to balance the two kinds of maximum products for fair comparisons ($\alpha = 0.95$ here).

Since the alignment technique here is rather simple, a revision could be done to enhance the performance of combination with homology based method. The widely used bioinformatics tool for sequence searching: Basic Local Alignment Search Tool (BLAST) is used to replace the original simple alignment. Take the unannotated *gag* sequence $S$ as the query sequence, and take all the positive NC sequences in the training set as the subject sequences. Then the NC sequence that could produce the most significant alignment results indicates the area most likely to be an NC in $S$. The locations of the putative initiation site and termination site after combination with BLAST (*blastp* here since the sequences are protein sequences) are shown as follow:

9 $$\left\{ \begin{aligned} & \mathop {\arg \max \{ \max }\limits_{m,n} D_{P} /\alpha ,\max A_{p} \} , \, if \, P_{M} \ge \beta \hfill \\ & \mathop {\arg \min }\limits_{p,q} B_{E} , \, if \, P_{M} < \beta \hfill \\ \end{aligned} \right.{ ,}$$

subject to

10 $$P_{M} = \max \{ \max D/\alpha_{P} ,\max A_{p} \}$$

Here $\min B_{E}$ is the minimum E-value produced by *blastp*. The subsequence between the $p{\text{th}}$ and the $q{\text{th}}$ amino acid is the corresponding area that produces the minimum E-value. $\beta$ is the threshold value that determines the selection of prediction results.

### Performance assessment

fivefold cross-validation was employed to assess the performance of the WSVM and RF models predicting the initiation sites and termination sites in this paper.

$G - mean$ under fivefold cross-validation was selected as the major performance evaluation measure. It also provide the basis for parameter selection of models. $S_{n}$, $S_{p}$, $ACC$ and $MCC$ were also calculated as a supplemental reference.

11 $$\left\{ \begin{aligned} & S_{n} = \frac{TP}{{TP + FN}} \hfill \\ & S_{p} = \frac{TN}{{TN + FP}} \hfill \\ &ACC = (TP + TN)/(TP + TN + FN + FP) \hfill \\ & MCC = \frac{(TP \times TN - FP \times FN)}{{\sqrt {(TP + FP)(TP + FN)(TN + FP)(TN + FN)} }} \hfill \\ & G - mean = \sqrt {S_{n} S_{p} } = \sqrt {\frac{TP}{{TP + FN}} \times \frac{TN}{{TN + FP}}} \hfill \\ \end{aligned} \right. \, {.}$$

where true positive ($TP$) and false negative ($FN$) are the number of positive samples that are predicted to be positive and negative respectively. Analogously, true negative ($TN$) and false positive ($FP$) are used to denote the number of negative samples that are predicted to be negative and positive respectively.

Among these evaluation measures, $G - mean$ and $MCC$ are better at providing a comprehensive view of the prediction performance, especially with our training dataset which has quantity imbalance between positive and negative data.

As with the performance assessment on prediction of entire NC proteins, leave-one–out cross-validation is applied. Each turn we pick out one *gag* sequence with NC annotation as the testing sequence and leave all others as the source of training samples. Then the above process is repeated until all sequences have been left out for a time as the testing sequence. The reason for not applying fivefold cross-validation here is to rule out random factors as much as possible, since fivefold cross-validation could generate different partition of datasets which may cause fluctuations in prediction performance. Such fluctuations could undermine the cogency of performance comparison between different methods*.* The prediction accuracy of the initiation sites, termination sites and entire NCs were calculated and compared.

### Detecting putative NCs in gags

When the NC predicting models are eventually built, the models could be used to search for more putative NCs in unannotated *gag*s. A fixed length sliding window is used to “scan” the unannotated *gag* sequences to find candidate NC initiation and termination sites. $L_{is}$ and $L_{ts}$ were set to equate with the length of the sliding window for convenience. The “WSVM & RF + SA” approach is adopted in the detecting process for speed and convenience. When $P_{M}$ is larger than the threshold $\beta$, its corresponding candidate NC boundary pair is predicted as a putative NC.

## Results

### Predicting Performance of the method

Prediction models based on strategy described above were built. Their effectiveness was also tested and shown below. (Prediction source code is available at SourceForge, with the download URL: https://sourceforge.net/projects/ncprediction/files/NCprediction.zip/download).

### Accuracy of the prediction of NC initiation and termination sites

The performance of the prediction models aimed at recognizing NC initiation and termination sites based on WSVM and RF were tested by fivefold cross-validation and shown in Table 1. From Table 1, we can find that the $G - mean$ values of initiation sites and termination sites are above 0.9900 and 0.9548 respectively. The $MCC$ values of initiation sites and termination sites are above 0.9735 and 0.9179 respectively. It indicates that both WSVM and RF models could generate satisfying results.

**Table 1:** Predicting performance of models applying WSVM & RF on initiation and termination sites of NCs.

NC Boundary Type	Algorithm	Sn	Sp	G-mean	Accuracy	MCC
NC Initiation site	WSVM	0.9869	0.9932	0.9900	0.9922	0.9735
	RF	1.0000	0.9974	0.9986	0.9978	0.9923
NC Termination site	WSVM	0.9227	0.9881	0.9548	0.9771	0.9179
	RF	0.9481	1.0000	0.9737	0.9913	0.9687

### Accuracy of the prediction of NC

All of the 77 retrovirus sequences collected with intact NC structures were used to test the predicting performance of different methods. Leave-one-out cross-validation is applied here to rule out random factors. We tested the performance of WSVM & RF, SA, *blastp* and their different combinations (please refer to S3 File for more details). The accuracy amount and rate of the prediction of initiation site, termination site and entire NC are shown in Table 2. We can find that the combination of machine learning methods and homology based methods could bring about better performance (prediction results of *blastp* is available at SourceForge, with the download URL: https://sourceforge.net/projects/leaveoneoutblastp/files/Blastleaveoneout.zip/download).

**Table 2:** Predicting performance of different methods on NCs.

Prediction method	NC sample set	Init Acc amount	Init Acc rate	Term Acc amount	Term Acc rate	NC Acc amount	NC Acc rate
*blastp*	All 77	56	72.73%	66	85.71%	47	61.04%
SA	All 77	61	79.22%	65	84.42%	51	66.23%
WSVM&RF	All 77	61	79.22%	61	79.22%	53	68.83%
SA + *blastp*	All 77	64	83.12%	65	84.42%	54	70.13%
WSVM&RF + SA	All 77	65	84.42%	64	83.12%	54	70.13%
WSVM&RF + *blastp*	All 77	66	85.71%	65	84.42%	56	72.73%
WSVM&RF + SA + *blastp*	All 77	67	87.01%	65	84.42%	57	74.03%
WSVM&RF + SA	66 with large P_M_	59	89.40%	59	89.40%	52	78.79%

It is also worth mentioning that the “WSVM & RF + SA” method performs better when there is $P_{M} \ge \beta$ ($\beta = 0.82$ here), which indicates that such method is reliable in detecting NCs in unannotated *gag*s. A self-consistency test was also performed with the “WSVM & RF + SA” method, 90.91% of the NCs could be predicted totally correct, the others are predicted with only slight deviations (please refer to S4 File for more details).

### Putative NCs

All of the 6041 available unannotated *gag* sequences were scanned with the “WSVM & RF + SA” model and 235 putative NCs in them were found (please refer to S5 File for more details, the putative NCs are marked in red).

## Discussion

### Conservative property of NC boundaries

**Figure 1:**
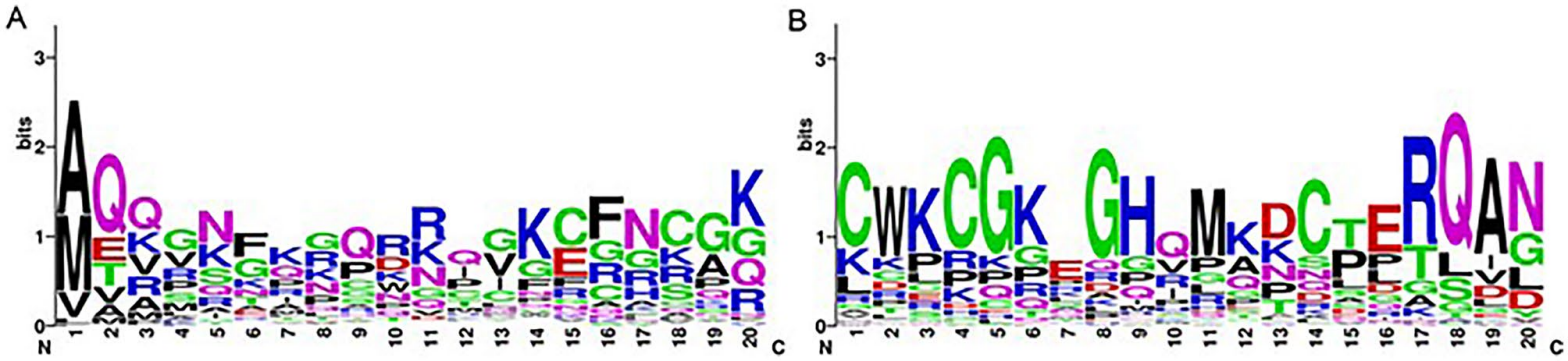
Motifs of residues adjacent to boundaries of NCs in ERV sequences. It shows motifs of surrounding residues of (**A**) NC initiation sites, (**B**) NC termination sites.

Motifs of sequences adjacent to origins and terminals of NCs in ERVs were generated based on WebLogo version 2.8.2 (http://weblogo.berkeley.edu/logo.cgi) and shown in Fig. 1. From Fig. 1, we can find that sequences adjacent to NC boundaries are quite conservative. This explains why satisfying predicting results could be generated from models built on starts and ends of NC.

### Feature importances

**Figure 2:**
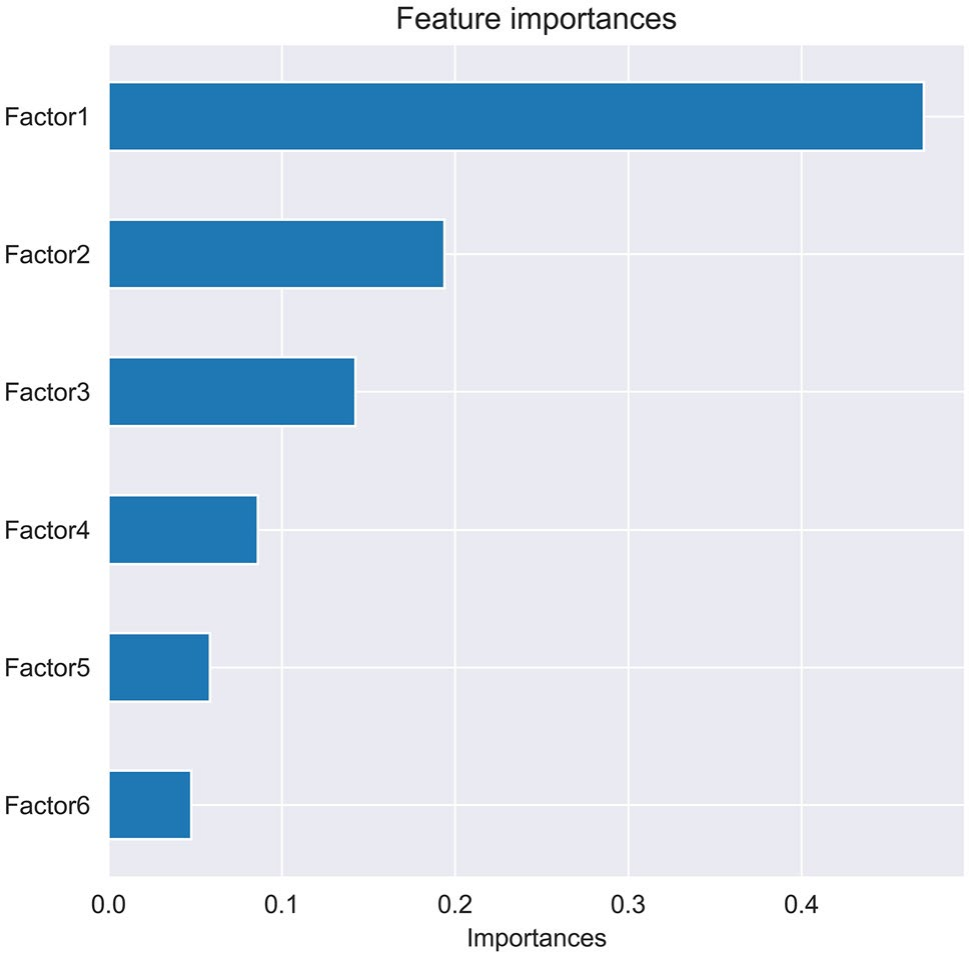
The feature importance of 6 factors of FASGAI.

The random forest classifier with its associated gini feature importance, allows for an explicit feature elimination^21^. Thus random forest classifier is utilized to calculate the feature importances of the FASGAI amino acid information. The feature importances of the 6 factors of FASGAI is shown in Fig. 2.

### Deep learning algorithm

**Figure 3:**
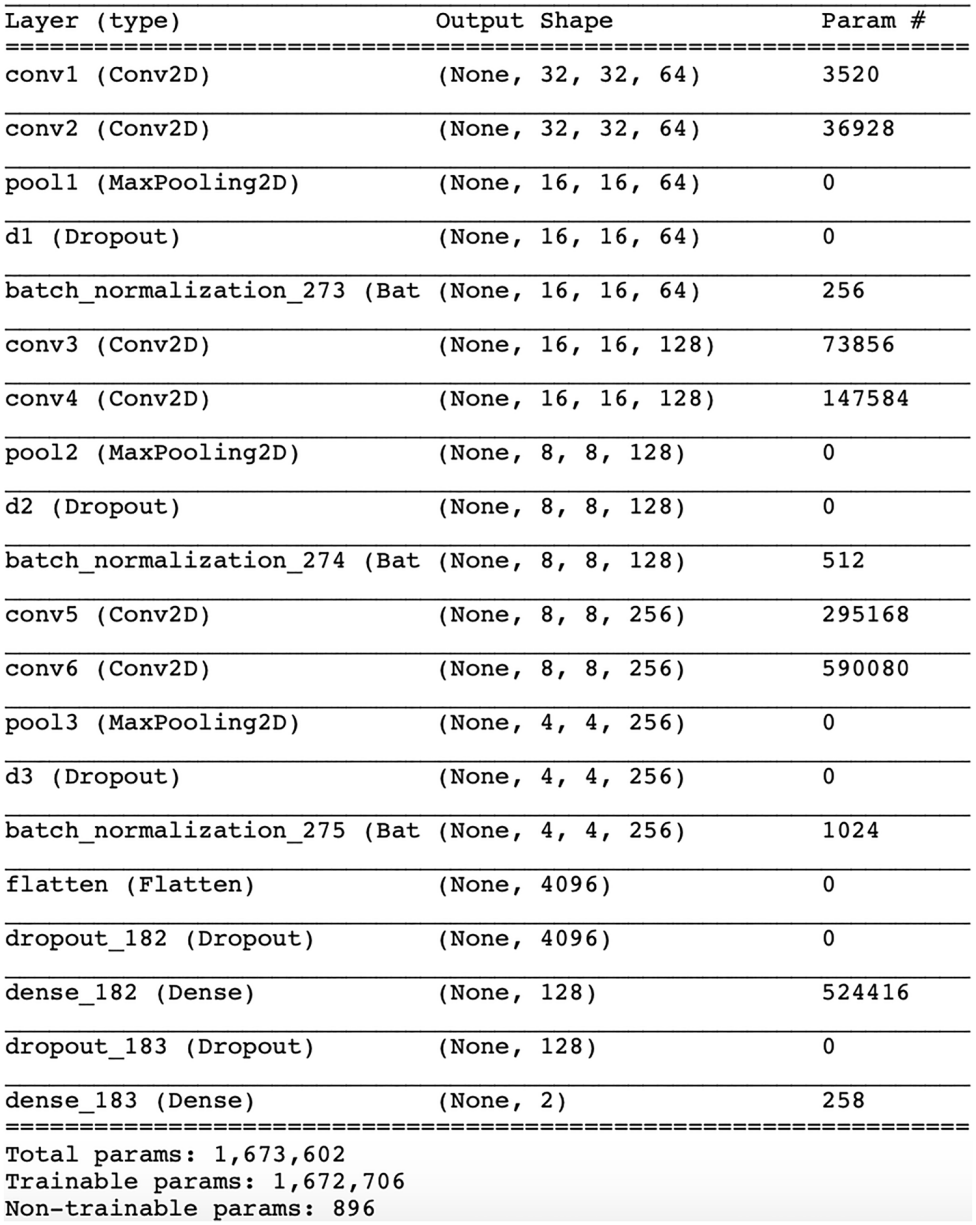
The model summary of the CNN model.

Along with the rapid development of deep learning these years, it is natural to try to use deep learning algorithms such as convolutional neural network (CNN) to solve the prediction of NCs. A CNN model was also built to solve the problem. The optimized model structure is shown in Fig. 3. The model contains only 6 convolutional layers, thus could be considered as a relatively simple CNN. However, the performance of the CNN model is not comparable with the“WSVM & RF + SA” approach, even though its training is much more time consuming. The detailed results were shown in Table 3, from which we could find that Sn rises along with the increase of fold number, while still not comparable with the Sn generated by WSVM or RF (shown in Table 1). The reason of this phenomenon is that deep learning algorithms contains more parameters to be iterated during the training process, but in this case, the sample size is not enough for the sufficient training of the parameters, so the“WSVM & RF + SA” approach suits better.

**Table 3:** Predicting performance of models applying CNN on initiation and termination sites of NCs.

NC Boundary Type	Fold Number	Sn	Sp	G-mean
NC Initiation site	fivefold	0.6195	0.9717	0.7759
	tenfold	0.6522	0.9413	0.7835
	leave-one-out	0.6957	0.9587	0.8167
NC Termination site	fivefold	0.5978	0.9348	0.7475
	tenfold	0.6304	0.9370	0.7686
	leave-one-out	0.6739	0.9565	0.8029

### Optimization of model parameters

Model parameters should be optimized until the model could bring out the best predicting performance. As with the WSVM & RF models, we adopted grid search to traverse the parameter space. The parameters that could bring out the highest value of *G-mean* were considered as the optimized combination of parameters. Since rerunning the model with one set of parameter combination for several times would compensate random factors with each other, another loop was added to the program to rule out arbitrary and capricious behaviours. As with the size of the sliding window in the putative NC detection process, the predicting performance of the “WSVM & RF + SA” method with different window lengths is tested and briefly shown in Table 4 (please refer to S6 File for more details). 16 was found to be an optimized value.

**Table 4:** Predicting performance of WSVM&RF + SA method with different window lengths.

WindowLength	Init Acc amount	Init Acc rate	Term Acc amount	Term Acc rate	NC Acc amount	NC Acc rate
1	0	0%	1	1.30%	0	0%
2	10	12.99%	11	14.29%	0	0%
3	39	50.65%	43	55.84%	32	41.56%
4	58	75.32%	64	83.12%	49	63.64%
5	43	55.84%	64	83.12%	35	45.45%
6	58	75.32%	64	83.12%	50	64.94%
7	58	75.32%	64	83.12%	49	63.64%
8	58	75.32%	64	83.12%	49	63.64%
9	55	71.43%	61	79.22%	42	54.55%
10	62	80.52%	61	79.22%	50	64.94%
11	63	81.82%	63	81.82%	53	68.83%
12	58	75.32%	63	81.82%	47	61.04%
13	63	81.82%	60	77.92%	51	66.23%
14	62	80.52%	63	81.82%	51	66.23%
15	61	79.22%	65	84.42%	51	66.23%
16	65	84.42%	64	83.12%	54	70.13%
17	60	77.92%	65	84.42%	50	64.94%
18	64	83.12%	64	83.12%	53	68.83%
19	61	79.22%	65	84.42%	51	66.23%
20	62	80.52%	65	84.42%	52	67.53%

### Evolutionary relationship analyses

**Figure 4:**
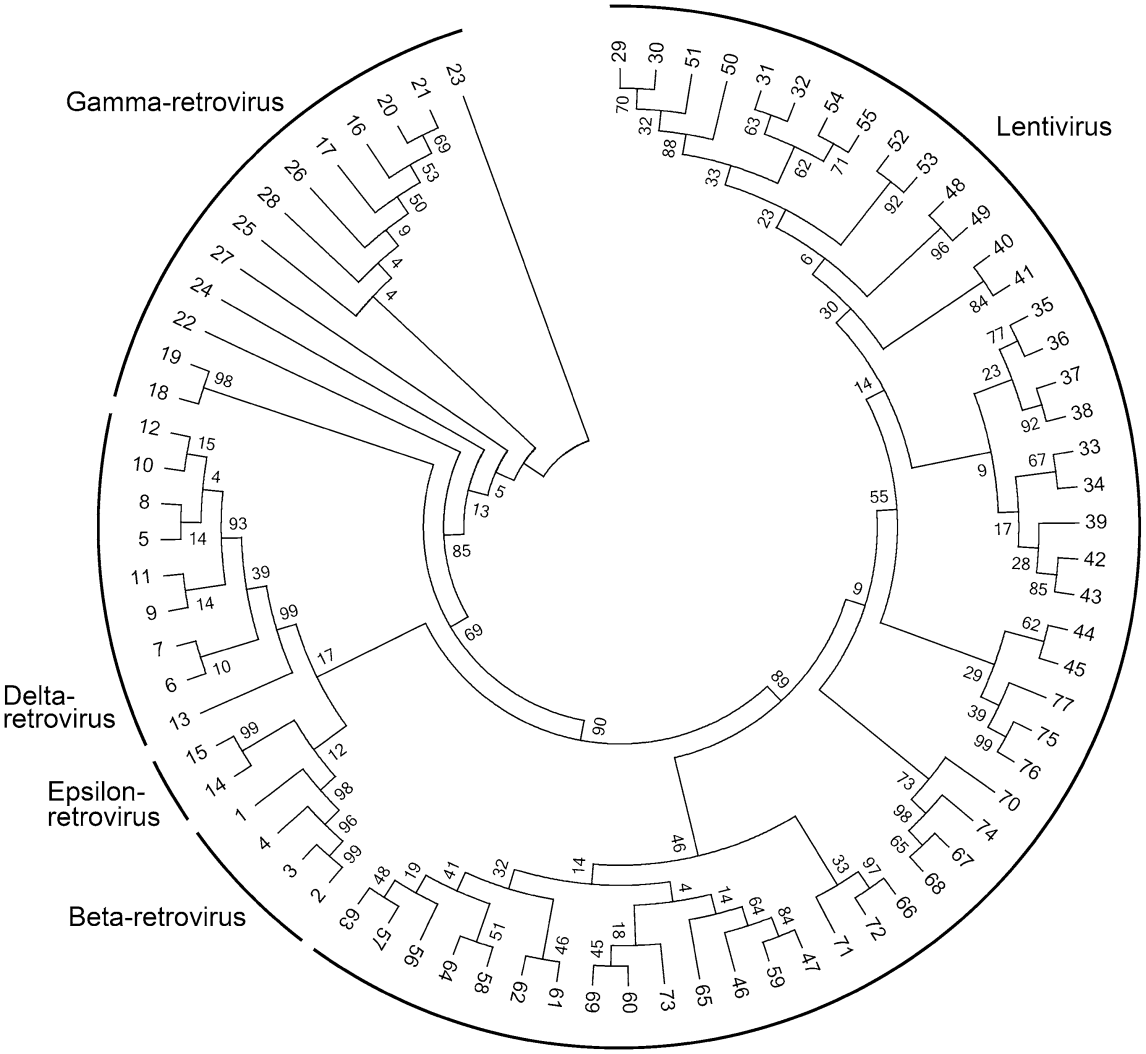
The evolutionary relationship of NCs in retroviruses. The leaf nodes denote annotated NCs in the benchmark dataset, and the edge lengths describe the phylogenic relationship between these nodes.

Evolutionary analyses were conducted in MEGA7^22^. The evolutionary history was inferred using the Neighbor-Joining method^23^. The evolutionary distances were computed using the Poisson correction method^24^ and are in the units of the number of amino acid substitutions per site. A comparison result between homology of NCs within genera and that of inter-genera is given in Fig. 4. It is obvious that NCs in the same genus are more homologous than that from different genera. This is identical with expectation and implies that genus-specified NC prediction methods could be brought up in the future to further enhance predicting performance when more annotated NCs are accumulated.

### Future outlook

The co-evolving information in the protein sequences is also verified to be useful for capturing the characteristics of proteins sequences^25–27^. Although these attempts were generally made in the area of protein–protein interactions (PPIs) instead of the prediction of functional elements, their basic idea to utilize co-evolving information do provide some enlightenment in the process of feature engineering, which might benefit us in our future research. Moreover, when more annotated NC sequences accumulate, the performance of deep learning algorithms could be improved since there would be enough information for the parameter iteration.

## Supplementary Information

Supplementary Information S1.

Supplementary Information S2.

Supplementary Information S3.

Supplementary Information S4.

Supplementary Information S5.

Supplementary Information S6.

Supplementary Information S7.

Supplementary Information S8.

Supplementary Information S9.

Supplementary Information.

## Data Availability

All data generated or analysed during this study are included in this published article (and its Supplementary Information files).
