# Supplementary material for: A computational method for predicting nucleocapsid protein in retroviruses
Source: Sci Rep. 2022 Jan 11;12:524. doi: 10.1038/s41598-021-03182-2 (PMC8752852; doi:10.1038/s41598-021-03182-2)
Supplement: Supplementary file 10 — Supplementary Information. [file 41598_2021_3182_MOESM10_ESM.docx]

**S1 File** Retrovirus sequences with NC annotations collected for research in this paper. (XLSX)

**S2 File** Details of the factor analysis scales of generalized amino acid information (FASGAI). (XLSX)

**S3 File** Details of the predicting performance of WSVM & RF, SA, blastp and their different combinations. (XLSX)

**S4 File** Details of the predicting performance of the “WSVM & RF + SA” method under self-consistency test. (XLSX)

**S5 File** Details of the results of NC detection by the “WSVM & RF + SA” method in unannotated gag sequences. (XLSX)

**S6 File** Details of the predicting performance of the “WSVM & RF + SA” method with different window lengths. (XLSX)

**S7 File** Details of “nucleocapsid protein retrovirus” search result in NCBI, packed in the GenPept (full) format. (GP)

**S8 File** Details of updated retrovirus sequences with NC annotations. (XLSX)

**S9 File** Details of source code and data of the research. (zip)
